# Modafinil in the rehabilitation of a patient with post-surgical posterior fossa syndrome: a lesson to be learned?

**DOI:** 10.1186/s40673-019-0105-6

**Published:** 2019-08-15

**Authors:** Emanuela Molinari, Maria Oto, Ashita Waterston, Natasha Fullerton

**Affiliations:** 1 Department of Neurology, Queen Elizabeth University Hospital, 1345 Govan Road, Glasgow, G51 4TF UK; 2 College of MVLS, School of Medicine, Dentistry & Nursing, University of Glasgow, University Avenue, Glasgow, G12 8QQ UK; 3 Scottish Epilepsy Centre, 20 St Kenneth Drive, Glasgow, G51 4QD UK; 4 Department of Medical Oncology, Beaton West of Scotland Cancer Centre, 1053 Great Western Rd, Glasgow, G12 0YN UK; 5 Department of Neuroradiology, Queen Elizabeth University Hospital, 1345 Govan Road, Glasgow, G51 4TF UK

**Keywords:** Posterior Fossa syndrome, Cerebellar mutism syndrome, Modafinil; behavioral

## Abstract

Disorders of the cerebellum may present with motor, cognitive, behavioral and affective symptoms. There is a growing interest in developing neuroanatomical models of symptoms generation that involve the cerebellum and the cerebello-cortical connections.

We describe an exciting first case report of successful use of Modafinil in an adult patient with post-operative posterior fossa syndrome. Following resection of a melanoma metastasis in the cerebellum the patient developed striking affective and behavioral symptoms in the form of withdrawn flat mood and disengagement. This neurobehavioral presentation severely impacted on his quality of life, independence, and ability to engage in the neuro-rehabilitative program. Pharmacological treatment with Modafinil ameliorated these emotional and behavioral aspects, and also fatigue. Treatment with Modafinil hence affected recovery and outcome for the patient.

To our knowledge, this is the first description of a successful pharmacological intervention in an adult with post-surgical posterior fossa syndrome and negative neurobehavioral presentation. Our findings illustrate the variability of the presentation of post-operative posterior fossa syndrome in adults, and the importance of delivering targeted treatment to maximize the benefits of neurorehabilitation.

The manuscript highlights the following points: 1. post-operative consequences currently under the wide umbrella of posterior fossa syndrome, can indeed manifest in adults; 2. a wide spectrum of neurobehavioral symptoms can occur, including a presentation with predominantly negative features; 3. the type of neurobehavioral presentation should guide the treatment choice with particular consideration of drugs that potentially modulate the cerebello-frontal connections; 4. Modafinil can be a candidate for effective treatment in presentations with predominantly negative behavioral symptoms.

## Background

Cerebellar lesions may manifest with symptoms across several domains, including neuropsychology, neurology, psychiatry, speech and language. These presentations are collectively described under the umbrella of “Posterior Fossa Syndrome” (PFS). The term PFS is often used interchangeably with Cerebellar Mutism Syndrome (CMS), a known pediatric consequence of posterior fossa surgery involving the cerebellum. The most recent definition of post-operative CMS characterizes it as a “delayed onset after surgery of speech and language impairment up to mutism, associated with emotional lability, and often accompanied by a variety of symptoms and signs such as motor deficits, swallowing difficulties, and coordination deficits” [1]. Thus, CMS, whilst sharing similarities with PFS, represents a more specific entity with two major diagnostic criteria (i.e. mutism and emotional lability) and with the key feature of severe speech and language disturbance.

The post-surgical consequences of the cerebellar injury in children were initially described in the 70ies [2, 3]. Over two third of primary brain tumors (BTs) in children over 1 year of age arise in the cerebellum or brain stem, and approximately a quarter of children undergoing posterior fossa surgery develop CMS [4]. Thus, CMS was initially considered unique to the pediatric population, likely as a logical conclusion from the frequent observation of CMS in children, and the predilection for the posterior fossa of primary pediatric BTs. The occurrence of CMS is a devastating experience for the child and the parents, often manifesting one to two days post-surgery, although with a more delayed onset in up to a quarter of cases [5]. Post-operative CMS is thought to be an acute side effects of surgery, which spontaneously improves over time, resolving on average in six to eight weeks [6, 7]. Despite mutism being transient and in spite of most symptoms improving over time, recovery is almost never complete [8]. For years, the acute presentation before surgery (i.e. hydrocephalus) and the complex post-surgical treatment that children with posterior fossa tumors receive (e.g. cranial radiotherapy) has masked the role that CMS plays in the child’s cognitive outcome. More recent studies have highlighted that developing post-surgical CMS is an independent risk factor for long-term cognitive decline [9, 10].

The increasing awareness of CMS and its consequences prompted clinicians and researchers to identify factors leading to, and factors predicting the development of CMS. However, results concerning tumor size, type and location [5, 7, 11–13], as well as different surgical approaches [14, 15], were conflicting. To date, medulloblastoma, brainstem invasion or compression, and younger age are considered risk factors for developing CMS [16]. From a surgical point of view, aggressive resection and vermian incision [17–20] are linked to increased risk of CMS. Nonetheless, the change in surgical practice towards sparing of the cerebellar vermis (i.e. telovelar approaches) did not see a convincing decline in its incidence. New efforts are focused on stratifying pre-operatively the individual risk for CMS [21].

Reports of adult patients with post-operative mutism also started to appear [22–26] as well as descriptions of a variety of sequalae involving cognition, behavior and affection following posterior fossa lesions and surgery [27–29]. Additionally, the range of presenting symptoms in children expanded with reports of isolated behavioral and affective disturbances following posterior fossa surgery, possibly linked to a vermian location of the lesion [30–32].

There remain several unanswered questions. Firstly, whether CMS, PFS and other presentations represent a spectrum of the same disease that, perhaps, manifests with a predominance of different symptoms across the life span. Secondly, whether post-surgical CMS itself manifests differently across age groups. The relatively low number of reports of CMS in adults may be multi-factorial, and only partly related to the low incidence of tumors in the posterior fossa compared to children. Adults may be less vulnerable to CMS due to a change in intrinsic susceptibility as the brain matures [22]. It is also possible that the reciprocal cerebello-cortical connections may be differently involved in specific functions during developmental stages, which may translate into age-specific symptoms. For example, with increasing age the presentation might move beyond mutism to a spectrum of neurobehavioral variants. Thirdly, whether different presentations carry a similar long-term cognitive outcome. Developing post-surgical mutism is linked with cognitive deficits later in life, which in turn impact on the individual’s independence and quality of life. It would be of utmost importance to determine if a post-surgical neurobehavioral presentation is associated with the same risk. Ultimately, there is no evidence on how and when to treat post-surgical consequences, such as PFS and CMS, to minimize their impact on physical and cognitive outcome.

Our case report aims to show that cerebellar post-surgical consequences in adult patients may present with isolated neurobehavioral symptoms and that such presentations may be limited to negative features (i.e. withdrawn behavior and affect). Additionally, we aim to show that, in our case, we obtained an exceptional recovery with the drug Modafinil, possibly through the selective enhancement of prefrontal-dependent cognitive functions [33]. Thus, we hope that the manuscript could enrich clinical practice by helping diagnosis and care of future patients presenting with isolated affective and behavioral disturbance following cerebellar surgery.

## Material and methods

We present a case report of an adult patient with severe emotional and behavioral disturbance in the absence of mutism (or severe speech and language impairment not attributable to other causes) following cerebellar surgery. Thus, we present a case which could be currently defined as a post-surgical “posterior fossa syndrome”. We question whether this may represent an adult-variant of the CMS spectrum. We describe the patient’s presentation, and the exceptional response to the drug Modafinil. We present the neuro-imaging and neuropsychology findings in order to discuss the underlying anatomy and pathophysiology.

## Clinical case

### History

The patient was a 44 years old male, right-handed native English-speaker. He had no past medical history of note, in particular no developmental delay, no delayed speech or language development, no cognitive or behavioral disturbance. He also had no cardio-vascular, neurological or psychiatric history of note, although he had a longstanding gambling addiction. The patient lived by himself and his family described him as a hardworking gentleman, chatty and cheerful, but at times with a short fuse and irritable. He had good friends and many interests.

**Fig. 1:**
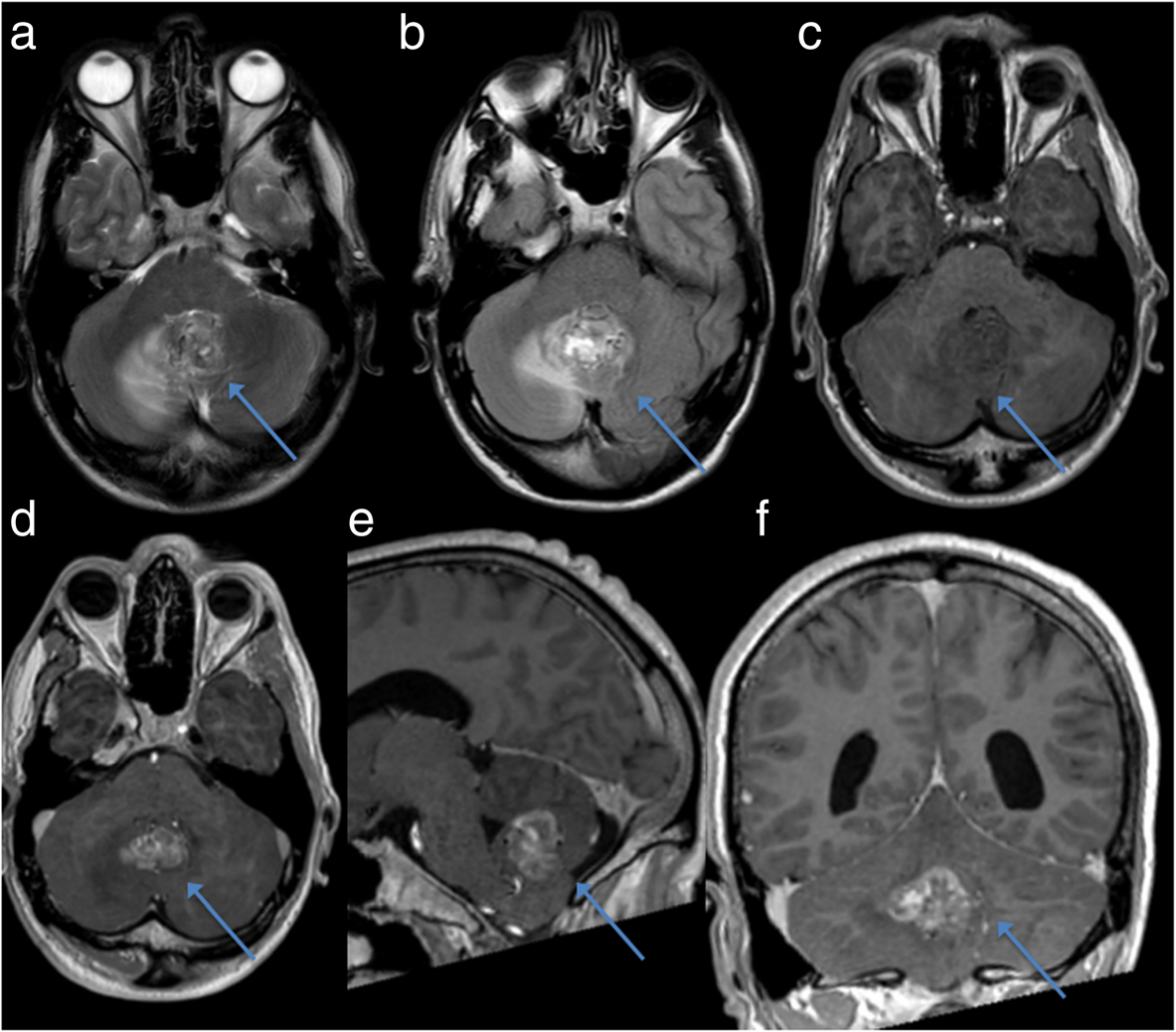
Pre-operative MRI brain demonstrating a heterogeneous, enhancing posterior fossa lesion with surrounding edema (blue arrow). 1**a**: Axial T2-weighted; 1**b**: Axial FLAIR; 1**c**: Axial T1-weighted pre-contrast; 1**d**: Axial T1-weighted post-contrast; 1**e**: Sagittal T1-weighted post-contrast; 1**f**: Coronal T1-weighted post-contrast

In 2017 he presented to hospital after three weeks of dizziness and daily headache, worse in the morning and associated with nausea. Investigations included a Computed Tomography (CT) brain, which revealed an avidly enhancing posterior fossa mass in the cerebellar midline and a small additional lesion in the right superior frontal gyrus. The subsequent Magnetic Resonance Imaging (MRI) brain showed a 22 mm by 31 mm by 25 mm intra-axial mass lesion centrally within the vermis with surrounding edema, extending more prominently into the right cerebellar hemisphere; with no features of hydrocephalus (Fig. 1).

He was commenced on Dexamethasone (16mg once daily), which led to improvement in his symptoms. Elective surgery for removal of the cerebellar lesion was planned two weeks later. At admission, the patient was hyperactive and slightly agitated with disrupted sleep, likely as a side effect of the steroids. The surgical technique involved a horizonal fissure approach with paravermian surgical incision of right and left cerebellar hemisphere. No ultrasonic aspiration was used during the surgery. A branch of the Posterior Inferior Cerebellar Artery (PICA) was entering the lesion and was electro-coagulated. There was no obvious swelling at the end of the surgical procedure and no clear hemorrhage. Pathology results of the brain tissue showed malignant melanoma, BRAF V600e positive (sequence variant valine to glutamic acid translocation). The patient underwent further investigations including CT thorax, abdomen and pelvis, which was negative. He was then diagnosed with resected Stage IV melanoma T0N0M1c [34].

### Post-operative findings

**Fig. 2:**
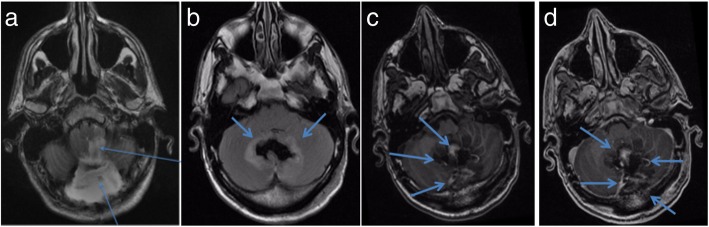
**a** axial T2-weighted, **b** axial FLAIR, **c** axial T1-weighted pre-contrast and **d** axial T2-weighted post contrast imaging demonstrate resection cavity with some blood degradation products and early post-operative enhancement; edema surrounding the resection cavity; contusion and edema left tonsil; and pseudomeningocele (blue arrows)

The immediate postoperative course was uneventful. The post-operative MRI (Fig. 2) showed no enhancing residual tumor in the posterior fossa. There was high Fluid-attenuated inversion recovery (FLAIR) and Diffusion-weighted Imaging (DWI) signal surrounding the resection cavity, more prominent in the left cerebellar hemisphere and left tonsil, suggesting some post-operative contusion and ischemia. A pseudomeningocele associated with the posterior fossa surgery was noted.

Clinically, on day one after surgery the patient was lethargic but rousable. On day two he was drowsy and obeying simple commands. On gross neurological examination, there was no cranial nerve or eye movement deficit, and no hypotonia or weakness. In the following days the patient tended not to interact, had moderately preserved vigilance with fluctuating drowsiness, and when prompted complained of headache, nausea and dizziness. Improvement in the patient’s level of alertness brought to light the consistent changes in his mood and behavior with predominant negative features (i.e. flat mood, reduced interactions, withdrawn affect). He showed substantially decreased voluntary motor activity and general apathy with disengagement. The patient usually lay with his eyes closed curled up in bed in a fetal position. There was no crying or whining. Auditory-verbal comprehension was intact for daily language use. His interactions were minimal. He was dismissive and abrupt with medical staff, but he was not aggressive. He replied to questions only when prompted to do so, and only spoke spontaneously to family members. Communication was characterized by monosyllables or two-word sentences without articles and determinants. His ate very little in spite of being able to swallow safely. At that time the patient’s weight was 54 kg with a height of 1.7 m (BMI 19). Due to the persistence of the behavioral change, a neuropsychology assessment was requested one month post-surgery. The patient was unable to comply with a structured assessment due to his poor interaction, poor engagement and lack of motivation. However, the psychologist noted the following features: 1) stark contrast with the patient’s behavior prior to surgery; 2) the patient required significant prompting to complete any task and this prompting led to the patient being agitated; 3) the patient had insight into his mood; 4) the patient had insight on discussions with medical staff that caused him annoyance; 5) rigidity and perseverance of thinking on selected topics that caused the patient anxiety; 6) sleep disturbances due to “mind racing”.

A repeat MRI brain showed an increase in size of the frontal melanoma metastasis and he commenced targeted anti-cancer therapy with Debrafanib and Tramatenib. Forty days post-surgery he deteriorated with increasing drowsiness, headache and nausea. He required an urgent ventriculoperitoneal shunt (attached to Medtronic Strata valve sets) for a sub-occipital pseudo-meningocele, likely aggravated by recumbency, with developing hydrocephalus.

Post shunt insertion, the patient’s headache and drowsiness had improved, whilst his neuro-behavioral presentation with negative symptoms continued. His speech and language did not show progressive improvement. He was defiant to complete daily tasks that required his collaborations, such as taking medicines, sitting up, standing, engaging in any physiotherapy, but he was otherwise submissive regarding major interventions, such as surgical decisions. Despite many attempts, he only managed to sit at the edge of the bed for 15 s before complaining of headache, dizziness and nausea. He did not show significant ataxia limiting his mobility whilst fatigue continued to increase. His day-to-day physical performances were not consistent and at best, he managed to walk from the bed to the toilet with assistance for balance. Despite stabilization and response to treatment of the right frontal lesion, his general health deteriorated with a progressive loss of weight due to decreased food intake. He lost 8 kg in four months (BMI 16). Attempts to support his nutrition via a nasogastric tube were overall unsuccessful, with the patient repeatedly pulling out the tube. At that point, there were concerns about his capacity to consent, he did not seem to retain information and showed little insight into his physical health. A decision to place a gastrostomy was made under the Adult With Incapacity (AWI) act, almost five months post initial surgery. The family was supportive of the decision and the patient did agree to have the procedure. The improved nutritional intake did not change the patient’s behavior or fatigue. Most days he refused to work with the physiotherapists and even when he agreed to it, the sessions lasted a few minutes at most.

### Follow up

Four months after the initial surgery, the patient was referred to a Neurologist with an interest in brain tumors and posterior fossa syndrome (author EM). The family members were present at the visit. His collaboration and the neurological examination were limited. He showed fatigue within a few minutes, verbal production was minimal with short sentences, slow, mildly dysarthric, slightly slurred but comprehensible. His mood was fluctuating and he showed some emotions when crying about his inability to be discharged home. Finger-nose test was slow but with no dysmetria. Muscle tone was normal. Gait was not tested at that time. In conjunction with the Psychiatrist (author MO), the patient was started on Sertraline, an antidepressant drug of the family of selective serotonin reuptake inhibitors (SSRIs), at a dose of 50 mg daily. There was no improvement after two months. Given the decline of the patient’s general health and the risk of missing the window when rehabilitation may be beneficial, the Neurologist (EM) and the Psychiatrist (MO) discussed a further medication trial. The patient was started on Modafinil (seven months after the initial surgery) with an increasing dosing scheme up to 100 mg twice daily.

**Fig. 3:**
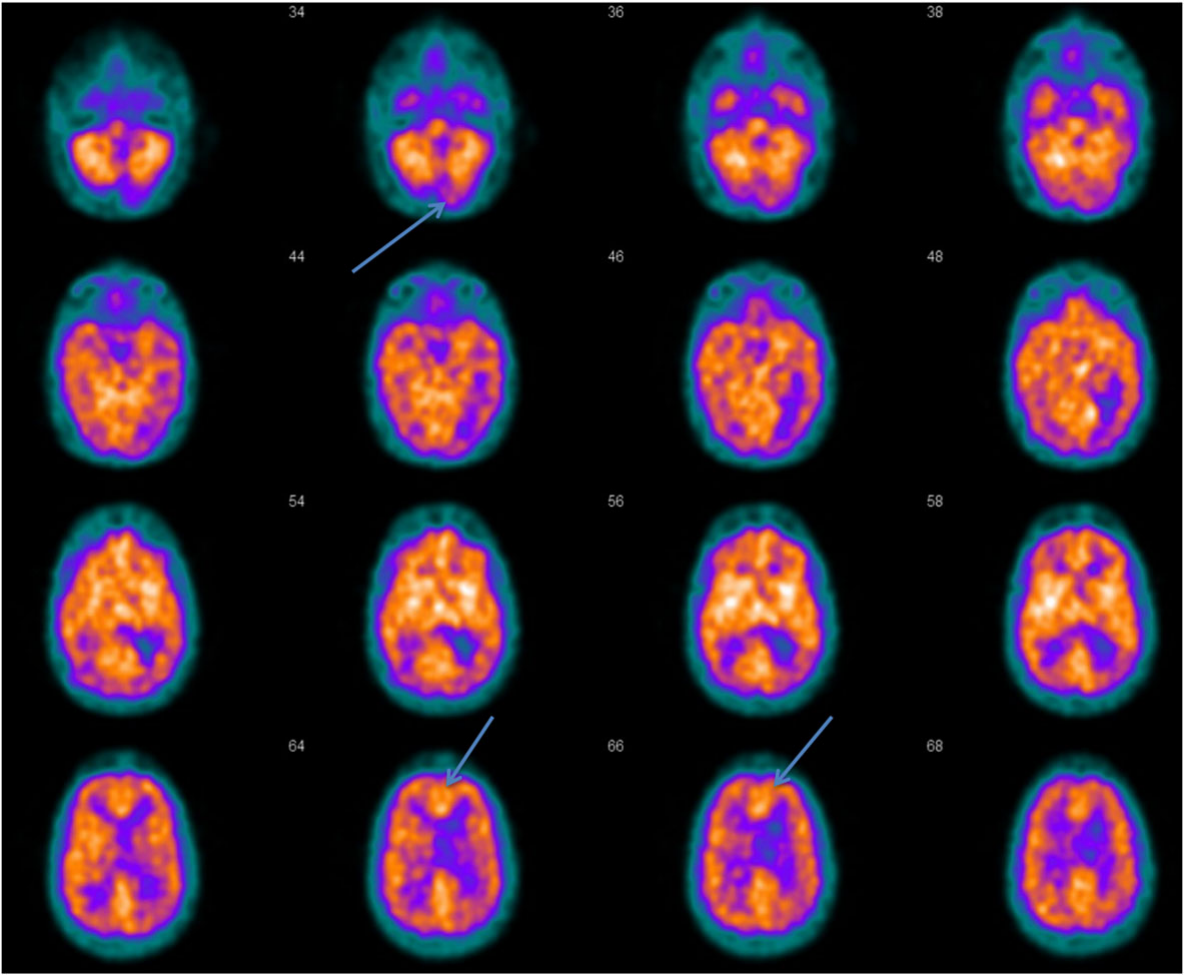
99 m Tc- HMPAO Perfusion SPECT scan showing focally increased perfusion at the level of the anterior cingulate (visualized on the last row, blue arrow). The blue arrow in the first row shows absent perfusion in the posterior fossa, as expected post-surgical finding

In the first week of starting Modafinil, the patient showed impressive improvements in all areas of evaluation, and progressively continued to recover. Specifically, he engaged in conversations not only with the family, but also with members of the hospital staff. He expressed himself with short conversations, which were not characterized by the previous child-like grammar. He engaged with the physiotherapists, he sat on a chair and walked down the corridor of the hospital with assistance. He started having short sessions of physiotherapy of approximately 10 min each, and continued to increase their duration with concomitant progressive reduction of the recovery time in between. He ate by himself and stopped using the gastrostomy tube completely after two weeks, with subsequent tube removal. He did not develop side effects related to treatment with Modafinil, and in particular he did not develop mania or psychosis. Although not specifically measured, the patient did not manifest autonomic-related symptoms such as heart rate or blood pressure issues. A perfusion HMPAO SPECT (single-photon emission computed tomography) CT perfusion scan of the brain during this recovery phase (two and a half months after starting Modafinil) showed expected reduced perfusion within the cerebellar vermis consistent with post-surgical cerebro-malacia, and normal frontal lobe cortical perfusion. In addition, there was focal hyperperfusion in the anterior cingulate (Fig. 3). Follow up CT brain confirmed the frontal lobe metastasis to be stable on the maintenance regime of BRAF inhibitors.

The patient continued to improve and gain from his physiotherapy and rehabilitation. Ten months post-surgery, and three and a half months following the start of Modafinil treatment, he had a staged return home (i.e. returning home at night). He was deemed fit for discharge home with rehabilitation support 12 months after the initial surgery, and five months after the start of Modafinil. Unfortunately, just a few days before his final discharge, neuroimaging showed progression of the metastatic lesions (progression of the frontal lesion with hemorrhage and new occipital lesion) and he became symptomatic with vomiting and focal motor seizures. Despite the progression of the cancer with unfavorable outcome, the patient maintained the benefits of Modafinil treatment on mood, behavior and engagement.

**Fig. 4:**
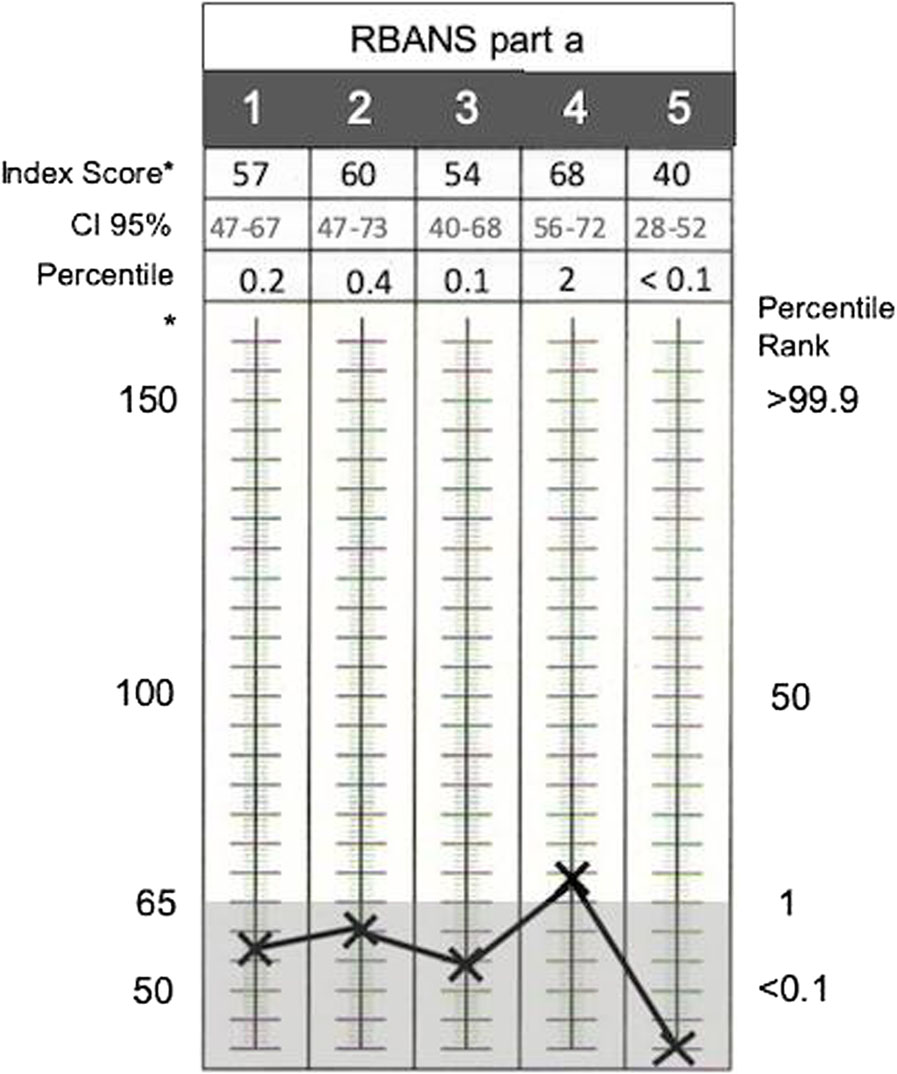
RBANS part a, visual summary of the patient’s performance. The patient’s scores are plotted in the standard assessment chart showing low score across the board (grey area highlights the percentile rank below 1° percentile) with relative strength in attention skills (forth column from the left). RBANS = Repeatable Battery for the Assessment of Neuropsychological Status. The columns numbered 1 to 5 represents the areas of assessment in RBANS part a. Columns from left to right: 1 = Immediate Memory; 2 = Visuospatial / Constructional; 3 = Language; 4 = Attention; 5 = Delayed Memory.

Other investigations at the time of the planned discharge included a neuropsychology assessment. In particular, the Repeatable Battery for the Assessment of Neuropsychological Status (RBANS) form a (Fig. 4), Hayling test and Trial Making A and B were performed. The patient’s performances revealed pretty low scores across the board of tests, but with relative strengths in attentional skills, visual screening ability and processing speed.

**Fig. 5:**
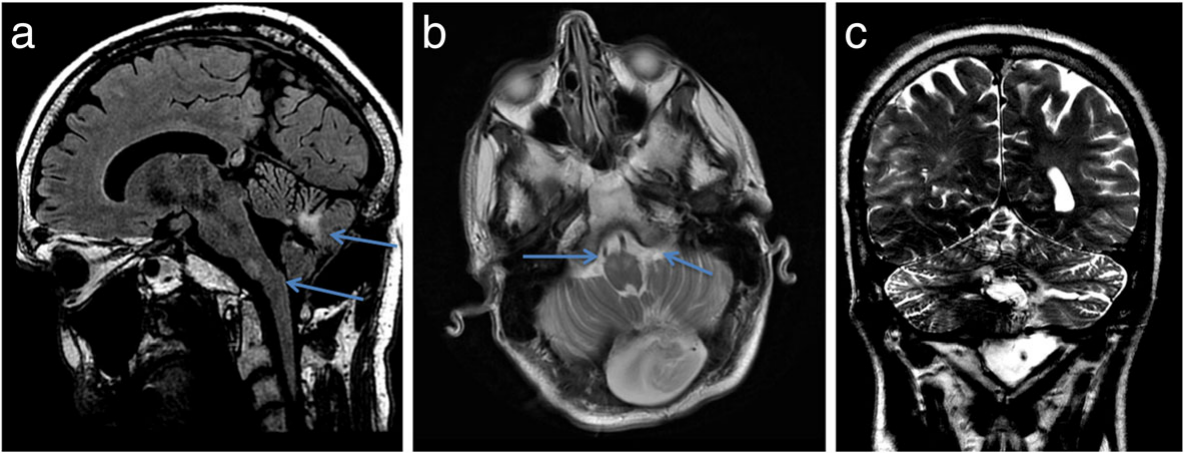
Sagittal FLAIR (**a**) showing (blue arrows) high signal affecting inferior olivary nuclei, and gliosis and malacia at the surgical bed with presumed damage to central tegmental tract and dentate nuclei. Axial T2-weighted (**b**) showing high signal medulla bilaterally, at the level of the inferior olivary nuclei. Post-operative pseudomeningocoele is also evident. Coronal T2-weighted (**c**) further highlights post-operative damage to cerebellum involving central tegmental tract and dentate nuclei with gliosis and hemosiderin deposition

The MRI brain at the same time showed bilaterally high T2-weighted and FLAIR signal in medulla oblongata at the level of the inferior olivary nucleus (Fig. 5), suggesting hypertrophic olivary degeneration as a result of bilateral damage of proximal efferent cerebellar pathways secondary to presumed peri-operative damage to the dentato-rubro-olivary pathways or Guillain-Mollaret triangle.

**Fig. 6:**
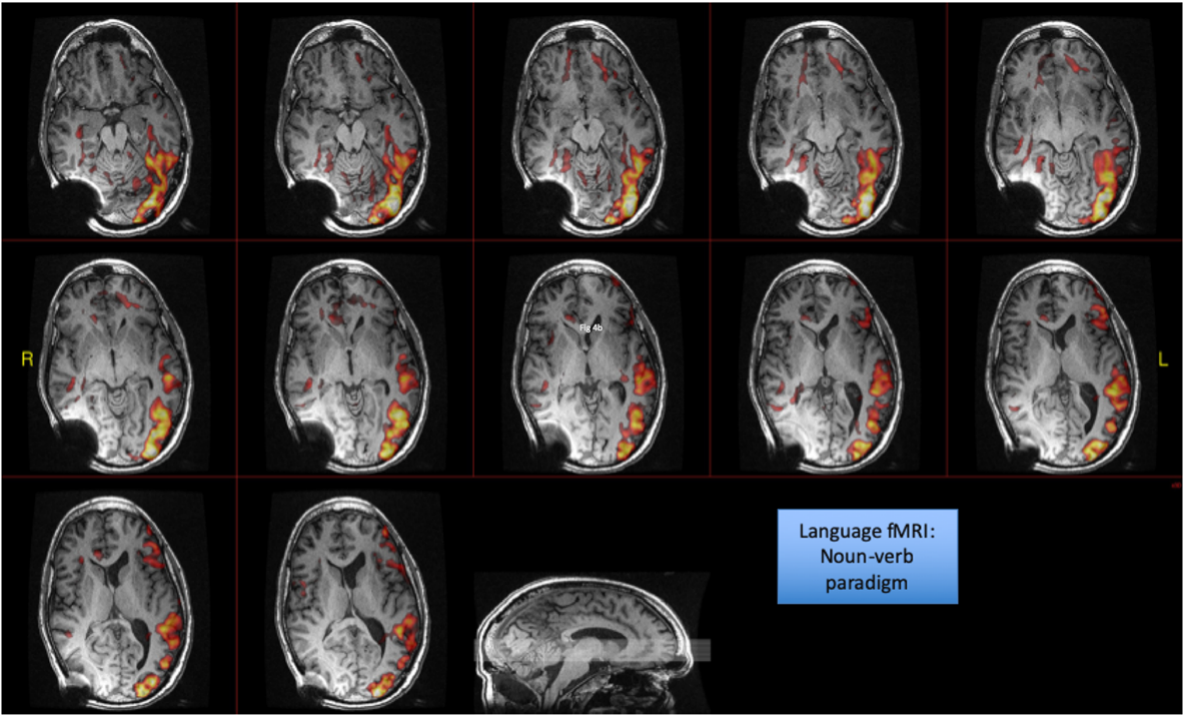
Language fMRI with word, noun-verb and stories paradigms was performed for language localization and lateralization. The underlying BOLD technique is susceptible to the artifact arising from the shunt. This demonstrated normal left hemispheric language activation in the frontal lobe, Broca’s area, and the superior temporal gyrus, Wernicke’s area

Functional MRI was also performed with three language paradigms for assessment of language centers and activation; this showed normal left hemispheric language activation in Broca’s and Wernicke’s area (Fig. 6).

**Fig. 7:**
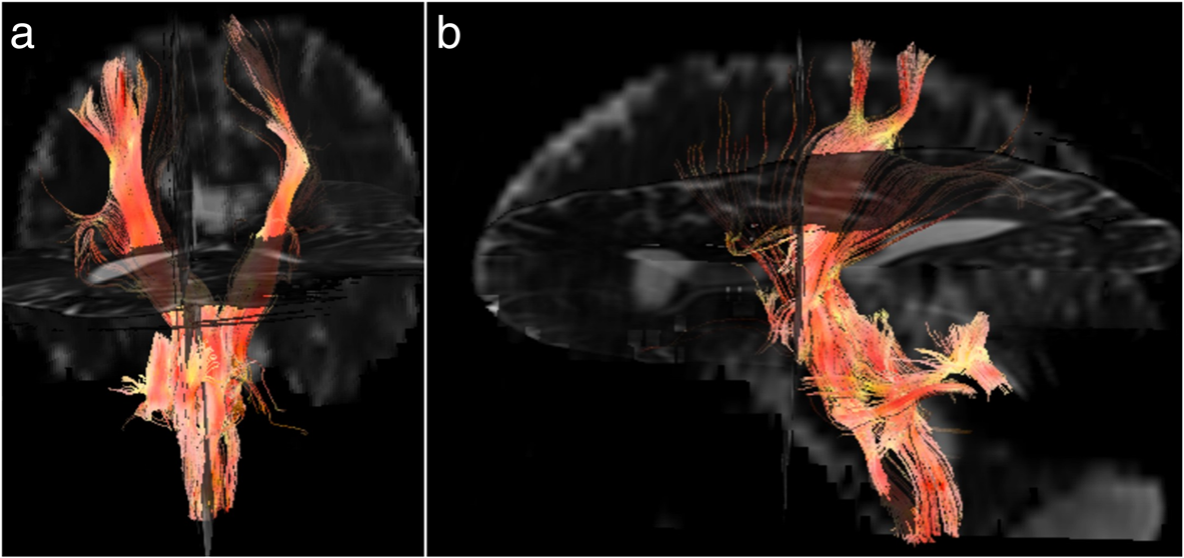
Diffusion tensor imaging was performed. A fractional anisotropy map was obtained and tracts were manually seeded. There was some degradation secondary to the shunt, however overall tracts, especially the corticospinal tract, remain intact. Coronal (**a**) and sagittal (**b**) views

Diffusion Tensor Imaging (DTI) (Fig. 7) was also performed, with fractional anisotropy map obtained and tracts seeded for tractography (Fig. 7). Although we could not see any gross abnormality of the tracts, the images were degraded by artifact arising from the right parietal shunt, limiting the value of the examination.

## Theory

There is growing evidence that the cerebellum acts as a master coordinator modulating cognitive, emotional and behavioral performances towards a relatively stable baseline [35–39]. The description by Schmahmann and Sherman (1998) [40] of a case series of patients with cerebellar pathology showing personality and behavioral changes, gave rise to the formal recognition of the cerebellar cognitive affective syndrome (CCAS) [29, 41]: a clinical syndrome linked to a variety of insults to the cerebellum and captured at a neuropsychological assessment by quantifiable deficits in executive, spatial, linguistic and affective domains. Recent studies of functional topography [42, 43] advanced the understanding of the non-motor role of the cerebellum, with the recent description of a pattern of distinct and multiple (triple) cerebellar representation in cognition and emotion task-processing. The connections between cerebellum and supratentorial brain, in particular through the cerebello-cortico pathways projecting to the pre-frontal and frontal cortex, have been implicated in several long-term effects of cerebellar injury [44–46] and mental health disorders such as schizophrenia. [47]. Additionally, the olivary nuclei [48, 49], the superior cerebellar peduncle [50–52] and the dentato-thalamo-cortical pathway [5, 12, 53–58] have been suggested as important anatomical substrates for CMS and PFS.

CMS and PFS do not have neuroimaging findings that are considered diagnostic and to date there is not a model able to predict the individual risk of developing either mutism or behavioral symptoms. It is possible that lesions in the posterior vermis produce dysregulation of affect [40, 59] possibly through the disruption of the dopaminergic system via the superior cerebellar peduncle and VTA, and the noradrenaline system via the locus coeruleus [28]. It has been recently suggested that the specific anatomical location of damage in the cerebellum (i.e. dentate nuclei versus vermian and fastigial nuclei) is critical to the predominance of certain symptoms (i.e. speech and/or behavioral abnormalities respectively) [60].

## Results and discussion

Our patient showed a striking change in his affect and behavior soon after posterior fossa surgery, which involved the cerebellar vermis but without splitting it. He showed marked speech and language impairment, although in our opinion he did not fulfill the criteria for mutism or speech impairment as per the most recent definition of CMS. The origin and extent of the speech and language involvement was difficult to determine due to the patient’s predominant withdrawn affect and disengagement. The fluctuation in his communication and the spontaneous and more florid communication and language used with family members made us consider that his speech impairment was mostly related to his behavioral presentation. The functional MRI performed almost one-year post-surgery revealed a normal pattern of hemodynamic activation for speech tasks, but we acknowledge that at that time the patient’s speech generation had normalized. Similarly, it is possible and even likely that the normal perfusion of the frontal cortex represents the normalization of the cerebral perfusion linked to the resolution of negative symptoms. This would be congruent with previous description of transient abnormalities in CBF during the mutism phase [6, 61, 62]. The patient’s behavioral symptoms affected the ability to perform a neuropsychology assessment for a prolonged time and we could not diagnose CCAS at onset. Although of limited value, his family did not notice any cognitive, affective or behavioral change prior to surgery. His neuropsychiatric presentation throughout showed a selective predominance of withdrawn and autistic spectrum behavior (e.g. lying in a fetal position) without the involvement of wider emotional and behavioral domains and/or the more typical alternation of positive and negative symptoms often seen in CCAS [63]. When the patient did manage to complete the neuropsychology assessment 11 months post-surgery, he, perhaps surprisingly, showed a relative strength in domains usually affected in CCAS. He could not complete the part B of the Trial Making test, whilst he still managed to complete the part A (53 s with no errors), scoring in the low average range. It has been suggested that patients with CCAS do struggle more with cognitive set shifting, which may be more reflected in the part B of the test [63, 64]. Additionally, our patient showed relative strength in domains such as line orientation, attention and language, thus making it difficult to diagnose CCAS [64]. Still, the deficits highlighted at the Trial Making test could be explained by the weakened modulation of the frontal lobe function from the cerebellum. With a single assessment, it is also difficult to assess how the pharmacological treatment has influenced the patient’s cognitive performance.

There have been attempts to distinguish CMS from the wider PFS umbrella. Post-surgical conditions are likely to require a different approach and management compared to other etiologies and could benefit from tailored strategies of risk stratification. The identification of risks factors before surgery could modify surgical decisions, for example the aggressiveness of the surgical resection.

We acknowledge that a single case report has significant limitations and somehow restricted learning value. Nonetheless, much advances have been made through single case observations and the authors believe that the presented manuscript provides unique insight for the diagnosis and care of patients with post-surgical cerebellar injury.

It is becoming evident that CMS can affect individuals across their life span. Recognizing the spectrum of CMS variants (i.e. behavioral disturbances without mutism) across age groups could better address the care of these patients. Impairment in emotional, behavioral and social skills domains, as well as cognitive domains, may negatively affect the neuro-rehabilitation and outcome in the short-term, and contribute to a diminished independence and quality of life in the long-term. Systematic cognitive assessment in patients who do not develop cerebellar mutism, and those displaying symptoms compatible with the current definition except for mutism, may reveal cognitive deficits that would otherwise go unnoticed. Long-term studies need to clarify whether these groups have the same long-term consequences, and may thus benefit from closer follow up and rehabilitation. It is possible that CMS, isolated behavioral presentation, and CCAS may represent different aspects of the neurological toxicity and sequelae induced by an injury to the cerebellum.

Importantly, we highlighted that emotional lability is characterized by a fluid presentation of emotional changes, which can manifest either with positive features (e.g. psychotic symptoms and agitation) or with negative features (e.g. withdrawn affect and autistic-spectrum symptoms) or a combination of them. The recognition of selective deficits as part of the same spectrum is of particular interest, especially in adults where clinicians are often not very familiar with the spectrum and condition of “posterior fossa syndrome”.

Those differing manifestations could require tailored interventions. Modafinil is a non-amphetamine central nervous system (CNS) stimulant and cognition enhancing drug. Modafinil’s effects include wake-promotion and neuroprotection [65]. It has been approved for the treatment of narcolepsy and it has shown potential benefits in Attention Deficit Hyperactivity Disorder (ADHD) [66, 67] and psychiatric diseases [68], such as depression [69] and schizophrenia [70]. Its exact mechanism of action is unknown [71] and to date no single receptor binding site has been isolated for the drug. The action of Modafinil seems to involve several neurotransmitters, either directly or indirectly, possibly with a varied effect in different areas of the brain [72]. Modafinil has been used on a few occasions in patients with brain tumors, specifically, patients with intrinsic primary brain tumors complaining of cognitive decline or fatigue [73, 74]. A phase II randomized clinical trial has been completed in 2016 by the University of South Florida to test Modafinil in children and young adults (6 to 19 years old) with primary brain tumors, with the primary outcome of changes in attention task (NCT01381718); results are awaited. Apart from this, Modafinil appears to have been rarely used in children with a brain tumor, mostly to relieve sleepiness in retrospective studies in a small number of patients [75]. Thus, to our knowledge, this is the first time Modafinil is used specifically to treat the neurobehavioral consequence of posterior fossa surgery in a brain tumor patient.

Modafinil has a short onset of action [76]. Our patient’s impressive improvement coincided with the start of Modafinil treatment. We have not identified any other factors that could have triggered such a clear-cut change in the recovery curve. He did not show a gradual recovery that could be attributed to compensatory mechanisms and plasticity. The increased cerebral perfusion in the anterior cingulate detected on the HMPAO-perfusion SPECT scan is consistent with previous studies on the site of action of Modafinil [77]. Our report has been limited by the timing of investigations available at our disposal. As we do not have a pre-treatment perfusion SPECT scan, it is possible that the noticeably increased perfusion in the anterior cingulate was already present, thus perhaps correlating more with the patient’s initial rigidity of thoughts and cognitive inflexibility [78]. In this scenario, we would have expected additional features of negative thoughts, obsessive-compulsive disorders and aggressive behavior, and a response to the serotonergic medication. Further research on CMS and PFS may investigate whether such finding, if reproduceable, represent a compensatory mechanism triggered by Modafinil in drug-respondent patients.

The use of Modafinil opens new opportunities and challenges. Among psychostimulants, the authors chose Modafinil since they had previous experience with its use. Other cognitive enhancing drugs, such as Methylphenidate, share the activation of brain areas such as the anterior cingulate and prefrontal cortex [79] and thus they may prove equally or perhaps even more beneficial. More studies are needed to highlight their specific effect on behavioral pathways and how their preferential activation of specific brain areas may influence the behavioral response. Similarly, it has to be carefully considered that such drugs may expose patients to a small risk of psychosis and mania [70, 80–82].

## Conclusion

We conclude that our patient developed post-surgical cerebellar neurobehavioral symptoms without mutism. We suggest that this may represent a variant of CMS sharing the same anatomical substrate. There is no guideline on whether patients with CMS and its variants may benefit from pharmacological approaches and cognitive behavioral interventions to effectively manage mood and behavioral changes. Short-term pharmacological intervention may maximize the engagement in both cognitive-behavioral and physical therapies. There are some case reports of successful use of antipsychotic such as Quetiapine [83–85] and of effective cognitive rehabilitation [86] in patients with a post-surgical neuropsychiatric and neurobehavioral presentation with positive symptoms. To our knowledge, there is a lack of reports on drug trials for the negative counterpart. This leaves patients at risk of missing the window of maximal benefit of rehabilitation. Our case report suggests that the use of Modafinil may prove beneficial in the post-operative period for negative neuro-behavioral features, and may allow the patient to engage with rehabilitation, thus maximizing the recovery and improving long-term independence. More systematic research is needed on the neurobehavioral presentation of cerebellar insult across the life span. We hope to encourage further reports of single adult patients with PFS, as these will constitute a comprehensive source for review of the clinical presentation, anatomical pathways and theoretical models. As clinical trials in rare syndromes may prove difficult, or not even be practically possible, case reports hold a high scientific value in advancing the field of knowledge and management by corroborating or rejecting initial findings.

## Data Availability

Not applicable.

## Abbreviations

5-HT: Serotonin (5-HT)
ADHD: Attention Deficit Hyperactivity Disorder
AWI: Adult With Incapacity
BMI: Body Mass Index
BOLD: Blood oxygenation level dependent;
BTs: Brain Tumors
CBF: Regional cerebral blood flow
CCAS: Cerebellar Cognitive Affective Syndrome
CMS: Cerebellar Mutism Syndrome
CNS: Central Nervous System
CT: Computed Tomography
DA: Dopamine
DTI: Diffusion Tensor Imaging
DWI: Diffusion-weighted imaging
FDG PET: Fluorodeoxyglucose positron emission tomography
FLAIR: Fluid-attenuated inversion recovery
GABA: Gamma-Aminobutyric acid
HA: Histamine
MRI: Magnetic Resonance Imaging
NA: Noradrenaline
PFS: Posterior Fossa Syndrome
PICA: Posterior Inferior Cerebellar Artery
RBANS: Repeatable Battery for the Assessment of Neuropsychological Status
SPECT: Single photon emission computed tomography
